# Analysis of the impact of pluronic acid on the thermal stability and infectivity of AAV6.2FF

**DOI:** 10.1186/s12896-024-00853-6

**Published:** 2024-04-25

**Authors:** Sylvia P. Thomas, Marcus M. Spinelli, Amira D. Rghei, Jordyn A. Lopes, Nicole Zielinska, Benjamin M. McLeod, Yanlong Pei, Wei Zhang, Bernard Thebaud, Khalil Karimi, Sarah K. Wootton

**Affiliations:** 1 Department of Pathobiology, University of Guelph, Guelph, ON N1G 2W1 Canada; 2 Department of Molecular and Cellular Biology, University of Guelph, Guelph, ON N1G 2W1 Canada; 3 The Ottawa Hospital Research Institute, Ottawa, ON K1Y 4E9 Canada

**Keywords:** Adeno-associated virus (AAV), AAV6.2FF, Pluronic acid, PF-68, Thermal stability, Cryostability, Intranasal, Lung transduction

## Abstract

**Background:**

The advancement of AAV vectors into clinical testing has accelerated rapidly over the past two decades. While many of the AAV vectors being utilized in clinical trials are derived from natural serotypes, engineered serotypes are progressing toward clinical translation due to their enhanced tissue tropism and immune evasive properties. However, novel AAV vectors require formulation and stability testing to determine optimal storage conditions prior to their use in a clinical setting.

**Results:**

Here, we evaluated the thermal stability of AAV6.2FF, a rationally engineered capsid with strong tropism for lung and muscle, in two different buffer formulations; phosphate buffered saline (PBS), or PBS supplemented with 0.001% non-ionic surfactant Pluronic F68 (PF-68). Aliquots of AAV6.2FF vector encoding the firefly luciferase reporter gene (AAV6.2FF-ffLuc) were incubated at temperatures ranging from -20°C to 55°C for varying periods of time and the impact on infectivity and particle integrity evaluated. Additionally, the impact of several rounds of freeze-thaw treatments on the infectivity of AAV6.2FF was investigated. Vector infectivity was measured by quantifying firefly luciferase expression in HEK 293 cells and AAV particle integrity was measured by qPCR quantification of encapsidated viral DNA.

**Conclusions:**

Our data demonstrate that formulating AAV6.2FF in PBS containing 0.001% PF-68 leads to increased stability and particle integrity at temperatures between -20℃ to 21℃ and protection against the destructive effects of freeze-thaw. Finally, AAV6.2FF-GFP formulated in PBS supplemented with 0.001% PF-68 displayed higher transduction efficiency *in vivo* in murine lung epithelial cells following intranasal administration than vector buffered in PBS alone further demonstrating the beneficial properties of PF-68.

**Supplementary Information:**

The online version contains supplementary material available at 10.1186/s12896-024-00853-6.

## Background

Adeno-associated virus (AAV) vectors are the leading platform for *in vivo* gene delivery. Currently there are seven Food and Drug Administration (FDA) or European Medicines Agency (EMA) approved AAV gene therapies including Glybera (2012), Luxturna (2017), Zolgensma (2019), Hemgenix (2022), Upstaza (2022), Elevidys (2023), and Roctavian (2023), all of which are based on naturally occurring serotypes [1–7]. While the majority of AAV vectors currently in clinical trials are derived from natural serotypes, engineered capsids with improved tissue specificity, transgene expressing capabilities, and immune evading properties are being developed and are now progressing toward clinical testing [8]. As with all new therapeutic modalities, vectors made using novel or engineered AAV capsids will need to undergo extensive product characterization as part of an Investigational New Drug Application (IND) to the FDA. This includes information about vector identity, safety, purity, potency, and stability, along with specifics about formulation, vialing, and vector stability under anticipated storage conditions [9, 10].

AAV has been shown to be a very stable vector, retaining infectivity over a range of pH’s and temperatures [11–14]. The standard temperature for long-term storage of AAV vectors is typically -70℃ or lower, where vectors retain their infectivity for at least two years [12, 15]. However, AAV vectors have been shown to remain stable at temperatures ranging between -80℃ to 55℃ for varying periods of time [13–15]. Analysis of AAV1 temperature stability revealed that the vector could be stored at 4℃ for up to four weeks without experiencing a significant loss of infectivity [14], and only after seven weeks at 4℃ did transducibility decrease by 20% [13]. On the other hand, incubation of AAV1 at 55℃ or higher for 20 minutes led to a statistically significant decrease in transgene activity [14]. The impact of freeze-thaw on AAV1 stability has also been investigated and it was found that after three freeze-thaw cycles the vector displayed highly variable transgene activity and by 10 freeze-thaw cycles there was a 40% reduction in transgene activity, suggesting that for short-term storage (e.g., a few weeks), it might be preferable to store the undiluted vector at 4℃ rather than it be subjected to multiple freeze–thaw cycles [13].

The type of storage buffer used can greatly impact the stability, infectivity, and aggregation of AAV vectors [16, 17]. Most clinical and commercial AAV buffer formulations include a surfactant such as Pluronic F-68 (PF-68), which aids in preventing vector adsorption to surfaces during manufacturing and product administration and may also reduce vector aggregation [18, 19]. In some cases, a cryoprotectant such as sucrose, sorbitol, or mannitol is added to stabilize AAV vectors during freeze-thawing. Since AAV serotypes differ in their chemical, physical, and thermal stability [13, 18, 20] buffer formulations need to be optimized for novel or engineered AAV capsids and should accommodate fluctuations in temperature that may be experienced during shipping, handling, and product administration [20–24].

AAV6.2FF is a rationally engineered triple mutant AAV with enhanced tropism for the lung and muscle [25]. Since AAV6.2FF is a novel vector, studies are required to fully characterize its stability profile. Since many vector cores and contract manufacturing organizations use PBS supplemented with 0.001% Pluronic F68 as a formulation buffer for AAV, we evaluated the impact of temperature and freeze-thaw cycles on AAV6.2FF expressing firefly luciferase (AAV6.2FF-ffLuc) formulated in PBS with or without 0.001% PF-68 to determine the impact of surfactant on the stability profile of this novel vector when exposed to temperatures ranging from -20℃, 4℃, 21℃, 37℃ to 55℃ for durations of 30 minutes to 2 weeks as well as repeated freeze-thaw cycles. The impact on infectivity was measured using an *in vitro* potency assay that measured luciferase transgene expression and the impact on AAV particle integrity was measured by quantitative PCR (qPCR) of encapsidated viral DNA. Both temperature and freeze-thaw treatments were compared to AAV6.2FF-ffLuc vector stored at the standard storage temperature of -80℃ to determine how these treatments influenced stability of AAV6.2FF in terms of transducing activity. Finally, we investigated whether buffer formulation impacted transgene expression of AAV6.2FF vector *in vivo* when administered intranasally to C57BL/6 mice.

## Methods

*AAV vector production.* AAV genome plasmids utilized in this study were engineered to contain a ubiquitous CASI promoter [26], the coding sequence for enhanced green fluorescent protein (eGFP; AAV6.2FF-eGFP) or Firefly luciferase (ffLuc; AAV6.2FF-ffLuc) followed by a woodchuck hepatitis virus posttranscriptional regulatory element (WPRE) and an SV40 polyadenylation signal. The entire cassette was flanked by AAV2 inverted terminal repeats (ITRs) and packaged into AAV6.2FF, which possesses three mutations, F129L, Y445F and Y731F in the capsid protein [25]. AAV6.2FF vectors were produced in adherent human embryonic kidney (HEK)293 cells (ATCC CRL-1573) and purified using heparin affinity chromatography as described previously [27]. AAV vector genome (vg) titers were determined by qPCR as described previously [27]. Briefly, viral genomic DNA samples were extracted using the Qiagen DNeasy Blood and Tissue Kit (cat no. 69506) and analyzed by qPCR using a TaqMan primer and probe set against the SV40 sequence:

SV40 p(A) Fwd Primer 5′-AGCAATAGCATCACAAATTTCACA-3′

SV40pA Rv Primer 5′- CCAGACATGATAAGATACATTGATGAGTT -3′and

SV40pA Probe: 5′-

/56-FAM/AG CAT TTT T/Zen/T TCA CTG CAT TCT AGT TGT GGT TTG TC/3IABkFQ/-3′.

(Integrated DNA Technologies), Luna universal qPCR master mix (New England Biolabs, M3003) and a LightCycler 480 thermal cycler.

### Aliquot preparation and temperature and freeze-thaw treatment regimen

**Fig. 1:**
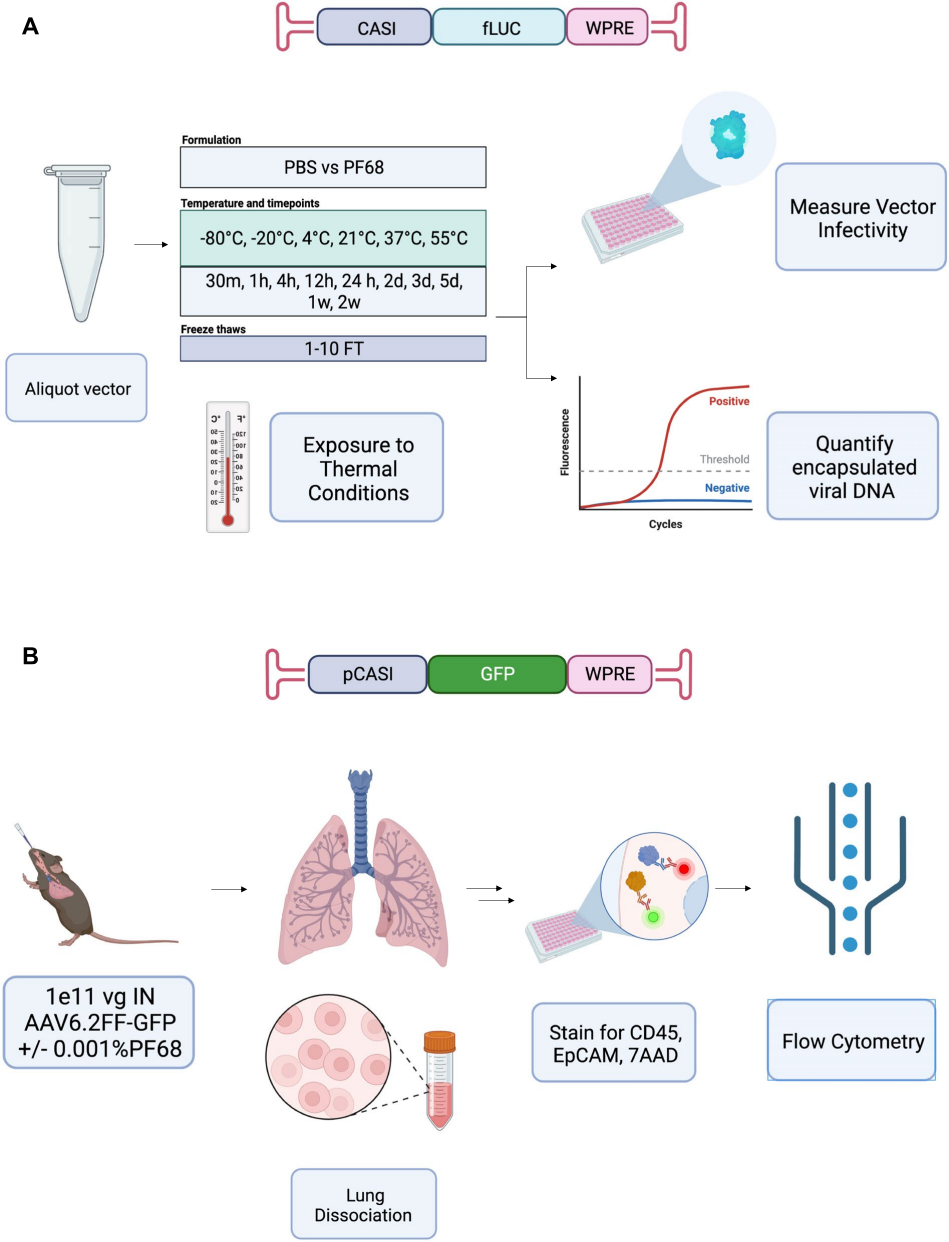
Overview of experiments assessing the influence of buffer formulation on the thermal stability and infectivity of AAV6.2FF. **A** *In vitro* assay design to evaluate the effect of buffer formulation on the infectivity and stability of AAV6.2FF-ffLuc at different temperatures and upon exposure to multiple rounds of freeze-thaw. AAV6.2FF-ffLuc vector was formulated in PBS or 0.001% PF-68-PBS and aliquoted in 1x10^9^ vg doses into 1.5 mL microfuge tubes. Samples (*n*=4) were subjected to incubation at -20℃, 4℃, 21℃, 37℃, and 55℃ for durations of 30 minutes (m), 1, 4, 12 or 24 hours (h) to 2, 3, or 5 days (d) or 1 or 2 weeks (w). Another set of samples (*n*=4) were subjected to 1 to 10 freeze-thaw cycles. All samples were evaluated for transducing activity in HEK293 cells (*n*=4) or for particle integrity by qPCR (*n*=3). **B** *In vivo* assay to investigate the effects of buffer formulation on the *in vivo* transducing properties of AAV6.2FF-eGFP. C57BL/6 mice (*n*=7) were intranasally administered 1x10^11^ vg AAV6.2FF-eGFP with or without 0.001% PF-68. Mice were euthanized 5 weeks post vector administration and lungs were harvested, dissociated, stained for CD45, EpCAM, and 7AAD and subjected to flow cytometric analysis

Aliquots of AAV6.2FF-ffLuc vector were diluted in PBS or PBS supplemented with 0.001% PF-68 to 2.5x10^10^ vg/mL aliquoted into 1.5 mL microfuge tubes made of high-clarity polypropylene (Fisher cat. no. 05-408-130). For the luciferase infectivity assay, 50 mL aliquots were prepared for each test condition (*n*=4). For the capsid integrity assay, 50 mL aliquots were prepared for each test condition (*n*=3). All tubes were stored at -80℃ until initiation of the experiment. The following treatment conditions were evaluated: -20℃, 4℃, and 21℃ were measured for a duration of 1 hour, 4 hours, 12 hours, 24 hours, 3 days, 1 week and 2 weeks. Additionally, samples were incubated at 37℃ for 1 hour, 4 hours, 12 hours, 24 hours, 3 days, 5 days and 1 week. Finally, timepoints for samples incubated at 55℃ included 30 minutes, 1 hour, 4 hours, 12 hours, 24 hours, and 2 days. An additional set of samples (*n*=3) were exposed to 1 to 10 freeze-thaw cycles. A freeze-thaw cycle involved removal of vector from the -80℃ freezer, thawing it at room temperature for 25 minutes and then returning the sample to -80℃ for a minimum of 24 hours until the next freeze-thaw cycle. Control samples stored at -80℃ were also maintained throughout the study as a point of reference. All the treatments described were performed for both buffer formulations. See Fig. 1A for an overview of sample treatment and analysis.

### Luciferase infectivity assay

HEK293 cells were seeded into 96 well tissue culture plates at 1x10^5^ cells per well and 24 hours later transduced with the treatment and control AAV samples at an MOI of 10,000. Three days later, the cells were harvested and lysed with the 2X Cell Lysis buffer provided in the kit. Lysates were analyzed for luciferase expression using the Pierce™ Firefly Luciferase Glow Assay Kit (ThermoFisher, cat. no. 16176) and the GloMax®-Multi Detection System plate reader (Promega) as per the manufacturer’s instructions

### Vector genome quantification

AAV samples were treated with 1 unit of Promega RQ1 RNase-Free DNase (M6101) for 20 min at 37℃ followed by heat inactivation at 75℃ for 15 minutes. Samples were then treated with 100mg of proteinase K (Invitrogen LSAM2546) at 50℃ for 60 minutes followed by heat inactivation at 95℃ for 30 minutes. Next, viral DNA was extracted using the Qiagen DNeasy Blood and Tissue Kit (cat no. 69506) and analyzed by qPCR using a TaqMan primer and probe set against the SV40 polyA sequence described earlier.

Genome copy numbers were quantified based on a 7-point, tenfold serial dilution standard curve generated with plasmid DNA.

### Animal experiments

All animal experiments were approved by the University of Guelph Animal Care Committee (AUP #4664) and were conducted in accordance with the Canadian Council on Animal Care. Five-week-old C57BL/6 female mice were purchased from Charles River Laboratories (Saint Constant, QC) and allowed to acclimate for one week prior to experimentation. Groups of 6-week-old mice (*n*=4) were anesthetized with 2.5% isoflurane and 1.0% oxygen and administered 1x10^11^ vg of AAV6.2FF-eGFP using a modified intranasal method of delivery as described previously [28]. See Fig. 1B for an overview of the *in vivo* experiment.

### Flow cytometry

Mice were euthanized at five weeks post-AAV administration by isoflurane overdose. After opening the thoracic cavity and nicking the renal vein, mice were infused with 20 mL of cold PBS in the right heart ventricle to perfuse the lungs. Next, 2 mL of 10U/ml of dispase II (Sigma, D4693) was instilled into the trachea and the trachea clamped with a hemostat to prevent leakage of the dispase. Lungs were then excised en bloc, placed in a 1 mL tube of dispase II and incubated at 37℃ for 6 minutes. Lungs were then transferred to gentleMACS C tubes (Miltenyi, cat. no. 130-096-334) and homogenized with the m lung 1.01 program on the gentleMACS Dissociator. Samples were filtered through 40 mm cell strainers (ThermoFisher, 22-363-547), centrifuged at 500xG for 5 minutes and the cell pellet resuspended in 1 mL of ACK buffer [8.29 g NH_4_Cl (0.15M), 1 g KHCO_3_ (10.0 mM), 37.2 mg Na_2_EDTA (0.1 mM) in 1 L MilliQ H2O] to remove red blood cells. After incubating for 5 minutes at room temperature, 5 mL of HBSS with Ca^2+^ and Mg^2+^ was added to neutralize the lysis buffer and samples were centrifuged. Cell pellets were resuspended in 200 mL of FACS buffer (PBS, 0.5% bovine serum albumin) and transferred to a U-bottom 96 well plate. The plate was centrifuged, supernatant removed, and samples resuspended in 1:200 anti-mouse CD16/32 antibody (BioLegend®, 101301) diluted in FACS buffer and 50 mL applied to each sample. After incubation at 4℃ in the dark for 20 minutes, 150 mL of FACS buffer was added and samples centrifuged again, and supernatant removed. Cells were resuspended in 1:100 dilution of PE/Cy7 anti-mouse CD45.2 (BioLegend® 109829) and APC/Fire™ 750 anti-mouse CD326 (Ep-CAM) (BioLegend® 118229) along with 5 mL of 7-AAD Viability Staining Solution (BioLegend® 420403) diluted in FACS buffer to a final volume of 50 mL per sample. Similar incubation and centrifugation steps were followed and once the supernatant was removed the samples were resuspended in 200 mL of FACS buffer and filtered through 100 mm filters into round bottom tubes. Analysis was performed on the FACS Canto II using FACS Diva Software. Compensations were conducted using single stained tubes containing cells from an untreated mouse and AAV-CASI-GFP-WPRE transduced HEK293 cells.

### Statistical analysis

Infectivity, capsid integrity, and *in vivo* transduction data were analyzed using Graph Pad Prism 9. A two-way ANOVA test was performed between the two buffer formulations in all exposure and treatment conditions, with the p-value threshold set at 0.05.

## Results

### AAV6.2FF formulated in PBS supplemented with 0.001% PF-68 is significantly more stable at lower temperatures than in PBS alone

**Fig. 2:**
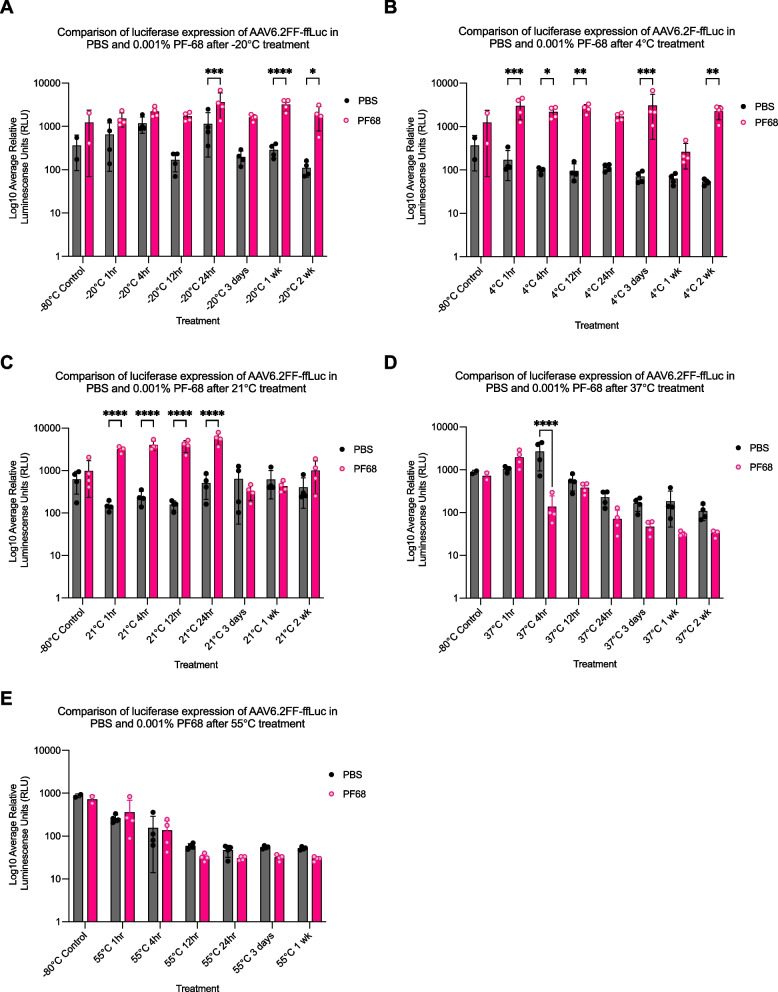
The effect of temperature on AAV6.2FF infectivity in different buffer formulations. Aliquots of 1x10^9^ vg of AAV6.2FF-ffLuc formulated PBS or PBS supplemented with 0.001% PF-68 were exposed to a range of temperatures and durations. Following treatment, vector aliquots were applied to HEK293 cells and luciferase expression measured in relative luminescent units (RLU) 72 hours later. The temperatures tested were as follows: **A** -20℃, **B** 4℃, **C** 21℃, **D** 37℃, or **E** 55℃. A two-way ANOVA test was performed between the two buffer formulations in all exposure and treatment conditions. *P ≤ 0.05, ** *P* ≤ 0.01, *** *P* ≤ 0.001, and **** *P* ≤ 0.0001

To investigate the impact of PF-68 on the stability of AAV6.2FF at different temperatures and when subjected to freeze-thaw cycles, samples of 1x10^9^ vg AAV6.2FF-ffLuc were formulated in either PBS or PBS supplemented with 0.001% PF-68 solution and subjected to the following temperatures: -20℃, 4℃, 21℃, 37℃, 55℃ and a control set at -80℃ for periods of time ranging from 30 minutes to 2 weeks. The potency of samples was measured by quantifying luciferase transgene expression after transduction of HEK293 cells (Fig. 2).

We observed that AAV6.2FF-ffLuc was equally stable in both formulations when stored for up to 12 hours at -20℃ but after incubation at -20℃ for 24 hours, 1 week and 2 weeks, the vector in 0.001% PF-68 buffer was significantly more infectious (*p*-value = 0.0015 and 0.0026, respectively) (Fig. 2A). Although there was no significant difference in AAV6.2FF-ffLuc infectivity between the two formulations at the 12 hour and 3-day time points at -20℃, there was a trend toward greater infectivity in the 0.001% PF-68 buffered samples (Fig. 2A and Figure S1A).

When AAV6.2FF-ffLuc was incubated at 4℃ for increasing periods of time, it was evident that the vector was not very stable at this temperature but that 0.001% PF-68 provided significant protection against loss of infectivity for both short (1 hour) and long-term (2 weeks) storage at 4℃ (Fig. 2B and Figure S1B).

Similar to incubation at 4℃, the infectivity of AAV6.2FF-ffLuc was reduced when the vector was incubated at 21℃ for increasing periods of time; however, 0.001% PF-68 provided significant protection against a loss of infectivity for up to 24 hours at 21℃, after which the protective effects of PF-68 were no longer evident (Fig. 2C and Figure S1C).

The protective effects of PF-68 were lost when AAV6.2FF-ffLuc was exposed to 37℃ temperatures for increasing periods of time (Fig. 2D and Figure S1D). In fact, after 4 hours incubation at 37℃, vector containing PF-68 was significantly less infectious than vector in PBS alone (*p*-value = 0.0003), which is opposite to what was observed when the vector was exposed to lower temperatures. Analysis of the average infectivity of AAV6.2FF over time supports this observation as the infectivity of the PF-68 containing vector drops significantly after 4 hours at 37℃ and remains lower than the vector in PBS alone, even as both formulations begin to diminish in their infectivity after 12 hours 37℃ (Figure S1D).

While it is unlikely that AAV vectors will be exposed to temperatures as high as 55℃, unless potentially during qPCR titration, we felt it was important to include a high temperature incubation as a putative positive control as well as to investigate whether there was a protective effect from the addition of PF68 at high temperatures. Incubation of AAV6.2FF at 55℃ led to a reduction in infectivity by 30 minutes with a continual reduction in infectivity over time (Fig. 2E). However, unlike at lower temperatures (e.g., 4℃ and 21℃) PF-68 did not appear to protect AAV6.2FF from a decrease in infectivity as the decrease in infectivity was comparable between the vectors formulated in PBS and PBS-0.001% PF-68. Although not significant, there was a trend toward the PBS-0.001% PF-68 containing vector being less infectious after incubation at 55℃ than the vector in PBS alone from 4 hours onwards (Fig. 2E and Figure S1E).

Finally, area under the curve analysis of mean luciferase expression levels over time after exposure of AAV6.2FF-ffLuc to -20℃, 4℃, 21℃, 37℃, and 55℃ clearly highlights the protective effects of PF-68 on preserving virus infectivity at low temperatures including to -20℃, 4℃, and 21℃ (Figure S2A).

### AAV6.2FF formulated in PBS supplemented with 0.001% PF-68 preserves particle stability over a range of different temperatures

We next wanted to investigate the impact of PF-68 on the integrity of the AAV6.2FF particle at different temperatures. We evaluated the integrity of vector particles using qPCR, reasoning that if we start with an equal number of virus particles, determined by vector genomes, any decrease in virus titer implies degradation of viral DNA. If the once-protected viral DNA became accessible or was released due to particle integrity breakdown from temperature changes, the DNase treatment that we perform prior to proteinase K treatment and viral DNA extraction, would degrade the exposed viral DNA. Based on this premise, the assay was used as a proxy for virus particle integrity.

**Fig. 3:**
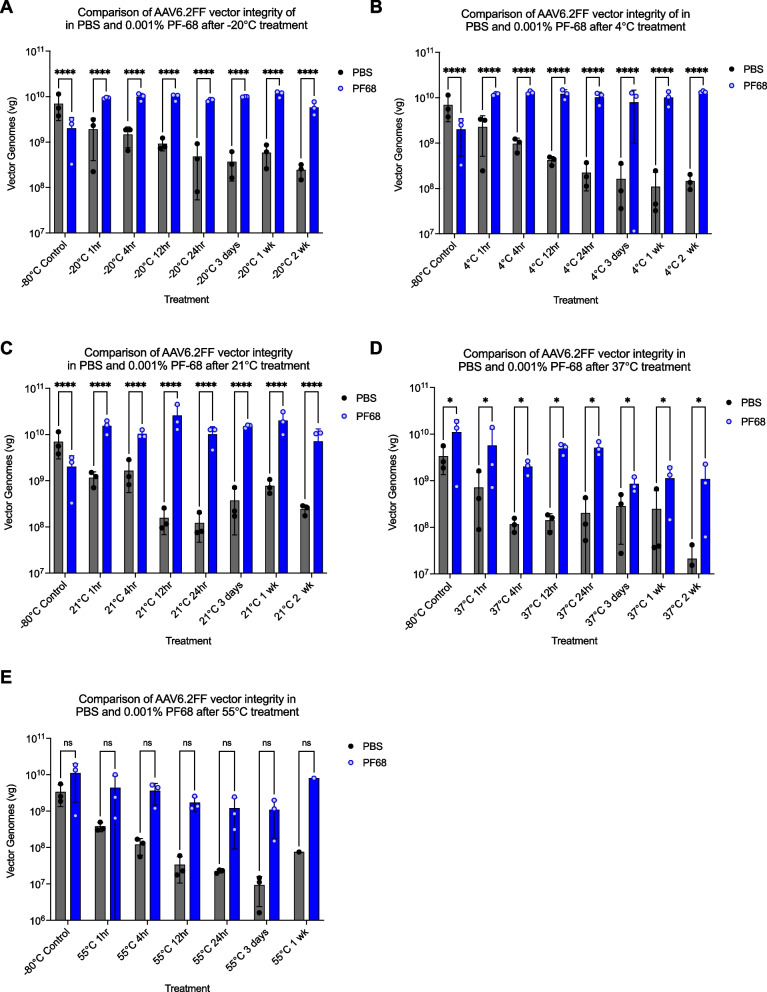
The effect of temperature on AAV6.2FF particle stability in different buffer formulations. Aliquots of 1x10^9^ vg of AAV6.2FF-ffLuc formulated PBS or PBS supplemented with 0.001% PF-68 were exposed to a range of temperatures and durations. Following treatment, viral DNA was extracted and quantified by qPCR. The conditions tested were as follows: **A** -20℃, **B** 4℃, **C** 21℃, **D** 37℃, or **E** 55℃. A two-way ANOVA test was performed between the two buffer formulations in all exposure and treatment conditions. ns (not significant), * *P* ≤ 0.05, and **** *P* ≤ 0.0001

When comparing total vector genomes after exposure of AAV6.2FF to -20℃, 4℃, and 21℃ for differing amounts of time, particle integrity was maintained in the samples formulated in 0.001% PF-68 and this was significantly better than vector formulated in PBS alone for all time points (Fig. 3A, B and C). Analysis of the mean vector genomes over time further support the notion that PF-68 helps stabilize the AAV6.2FF particle when exposed to temperatures ranging from -20℃ to 21℃ (Figure S3A-S3C). In contrast, the ability of PF-68 to preserve AAV6.2FF particle integrity began to wane at 37℃. Although AAV6.2FF vector formulated in 0.001% PF-68 retained significantly more intact virus particles after incubation at 37℃ for extended periods of time than vector formulated in PBS alone, the p value was * *P* ≤ 0.05 (Fig. 3D) compared to ****P ≤ 0.0001 for -20℃ to 21℃ (Fig. 3A-C). At higher temperatures such as 55℃, there was no significant preservation of particle integrity in the AAV6.2FF vector sample formulated in 0.001% PF-68, even after 30 minutes; however, analysis of the average vector genomes over time showed a trend toward greater particle integrity of the AAV6.2FF vector sample formulated in 0.001% PF-68 (Figure S2B).

### PF-68 preserves AAV6.2FF infectivity and particle integrity when exposed to multiple freeze-thaw cycles

**Fig. 4:**
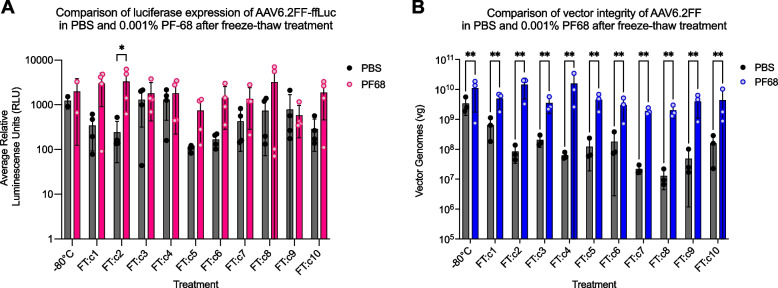
The effect of freeze-thaw on AAV6.2FF infectivity and particle stability in different buffer formulations. Aliquots of 1x10^9^ vg of AAV6.2FF-ffLuc formulated PBS or PBS supplemented with 0.001% PF-68 were exposed to 1 to 10 cycles of freeze-thaw after which the samples were either applied to HEK293 cells and luciferase reporter gene expression quantified 72 hours later **A**, or viral DNA was extracted and quantified by qPCR **B**. A two-way ANOVA test was performed between the two buffer formulations in all exposure and treatment conditions. * *P* ≤ 0.05, ** *P* ≤ 0.01

It is generally understood that AAV vectors denature, form aggregates, and lose infectivity when exposed to freeze-thaw cycling.[24] Here we subjected AAV6.2FF formulated in PBS or PBS supplemented with 0.001% PF-68 to 10 rounds of freeze/thaw (F/T) and the effect on AAV6.2FF transduction and AAV6.2FF particle stability tested as described above. The first significant effect on transduction was observed after the second F/T where AAV6.2FF formulated in PBS supplemented with 0.001% PF-68 was significantly more infectious than vector in PBS alone (*p*-value = 0.0253) (Fig. 4A). While there was considerable variability in transduction efficiency with subsequent freeze–thaw cycles, there was a trend toward reduced infectivity of the vector formulated in PBS compared to PBS supplemented with 0.001% PF-68 (Figure S4A).

In contrast to the infectivity data, AAV6.2FF particle stability was significantly increased when PF-68 was present, even after 10 F/T cycles (Figure S4B). This was demonstrated by the minimal fluctuation in AAV6.2FF vector genomes in the 0.001% PF-68 formulated samples over 10 freeze-thaws compared to the steady decrease in vector genomes in the PBS formulated samples (Figure S4B).

### AAV6.2FF formulated in 0.001% PF-68 had significantly higher transducing activity in the mouse lung compared to vector without PF-68

**Fig. 5:**
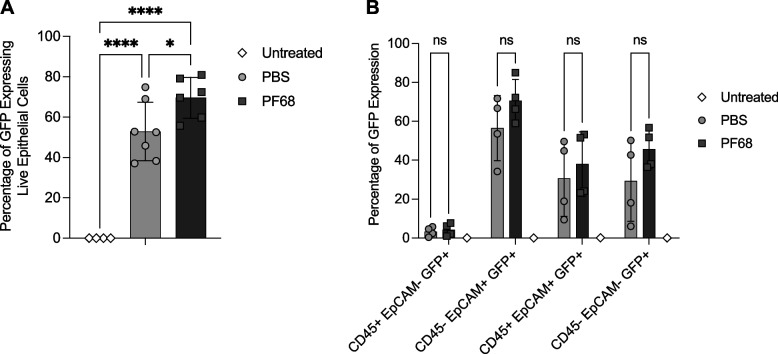
Impact of 0.001% PF-68 on the *in vivo* transduction efficiency of AAV6.2FF. Six-week-old C57BL/6 female mice (*n*=7) were intranasally administered 1x10^11^ vg of AAV6.2FF-eGFP formulated in either PBS or PBS+0.001% PF-68 and 5 weeks later, lungs were harvested and analyzed for GFP expression by flow cytometry. **A** The percentage of GFP expressing live epithelial cells (*n*=7) in either buffer formulation compared to an untreated control using two-way ANOVA. * *P* ≤ 0.05, **** *P* ≤ 0.0001. **B** Analysis of the different live cell populations (7AAD^-^, CD45^+/-^, EpCAM^+/-^) that were transduced by AAV6.2FF-eGFP (*n*=4)

Next, we investigated whether PF-68 would influence the *in vivo* transducing properties of AAV6.2FF, including tropism. Groups of 6-week-old C57BL/6 female mice (*n*=4) were intranasally administered 1x10^11^ vg of AAV6.2FF-eGFP with or without 0.001% PF-68. Mice were euthanized five weeks post AAV administration, and the lungs harvested for flow cytometry. After dissociation, lung cells were stained with antibodies specific for CD45 and EpCAM, and 7AAD viability dye and subjected to flow cytometric analysis. The percentage of live eGFP positive CD45^-^ EpCAM^+^ 7AAD^-^ lung epithelial cells, a primary target for AAV6.2FF, was significantly higher in the mice transduced with AAV6.2FF-eGFP plus 0.001% PF-68 (70.75%) than in mice transduced with AAV6.2FF-eGFP in the absence of 0.001% PF-68 (51.87%) (*p*-value = 0.0148) (Fig. 5A). Analysis of other cell populations revealed a trend towards increased numbers of eGFP positive cells, except for cells displaying the immune cell marker CD45 (Fig. 5B).

## Discussion

Surfactants such as Pluronic-F68 have been shown in several studies to be crucial for preventing adsorption of AAV vectors to surfaces during manufacturing and administration, as well as reducing vector aggregation.[17] For this reason, Pluronic-F68 is included in the formulation buffers of many AAV products. Of the 37 AAV products in clinical phase development and with health authority license approval, with known formulation composition, that were identified in a recent publication by Grossen et al. [17], 15 had in Poloxamer 188 (ranging from 0.0001% to 0.2% w/v) in their formulation buffer. Of these 37 AAV products, only two used an AAV6 serotype capsid, which is a close relative of AAV6.2FF, and of the two AAV6 products, only one included Pluronic-F68 (0.05% w/v) in the formulation buffer. Moreover, unlike the 37 AAV products listed in the publication by Grossen et al. [17], which were administered via intramuscular, intrathecal, intravenous, or subretinal routes, our AAV6.2FF vector will be delivered directly to the respiratory tract. Given that it is necessary to identify solution conditions that are both stabilizing and suitable for different routes of AAV administration [18], we embarked on these studies.

Here, we evaluated the thermal stability of AAV6.2FF when exposed to various temperatures and freeze-thaw cycles in PBS buffer with and without supplementation with the non-ionic surfactant PF-68. At temperatures ranging from -20℃ to 21℃, we observed relatively stable vector infectivity and particle integrity across all timepoints when PF-68 was present but not in samples without PF-68. Conversely, the stabilizing effects of PF-68 were no longer observed at 37℃ and 55℃; in fact, PF-68 may have destabilized the AAV6.2FF particle when incubated at these high temperatures for more than 4 hours. When exposing AAV6.2FF to multiple freeze-thaw cycles, we observed a significant drop in infectivity after the second freeze-thaw in the PBS only samples compared to the PF-68 containing samples. Additionally, PF-68 containing samples displayed less variability in infectivity between freeze-thaw cycles, especially for the first four cycles, and higher overall transducing activity compared to vector in PBS alone. Finally, there was reasonably good similarity between the results of the infectivity and particle integrity assays. Together, these data demonstrate that formulating AAV6.2FF in PBS containing 0.001% PF-68 leads to increased stability and particle integrity at temperatures between -20℃ to 21℃ and protection against the destructive effects of freeze-thaw. Our findings are consistent with previous work in which PF-68 was shown to prevent surface adsorption and aggregation, mitigate capsid rupture during freeze-thaws with AAV8 and AAV9, and improve stability when an AAV2 vector is administered surgically to the eye [19].

The fact that formulation of AAV6.2FF with buffer containing PF-68 showed increased stability and preservation of infectivity at temperatures such as -20℃ and 4℃, which are readily accessible storage temperatures in a clinical setting, means that it might be possible to store vector at -20℃ before it is thawed, or at 4℃ for short periods of time (e.g., 1 week) after thawing to avoid multiple freeze-thaw cycles. Moreover, it may be possible to revise cold chain requirements for storage and transportation of AAV6.2FF to those that are less costly and logistically demanding [29]. Recent developments in formulations for freeze-drying that maintain AAV potency for 24 months at 2-8℃, and film matrixes that increase long term vector stability at room temperature or 4℃ [21, 30, 31] are future directions that can be explored to simplify the storage and shipping conditions for AAV6.2FF.

The higher temperature conditions evaluated in this study were selected to represent conditions in cell culture and following *in vivo* administration (37℃) as well as temperatures used for heat inactivation, for example for adenovirus that is sometimes included if AAV vectors are being evolved through DNA shuffling or in some cases for large scale production [32, 33]. We observed a sharp decrease in the infectivity of PF-68 containing vectors relative to vectors without PF-68 four hours after incubation at 37℃, and this trend toward lower infectivity in the presence of PF-68 continued for the duration of the time course. While PF-68 is not being considered as an additive during AAV vector production, vectors are exposed to temperatures of 37℃ after *in vivo* administration. Thus, it is possible that the presence of PF-68 could impact *in vivo* transduction efficiency of AAV, although the rate at which AAV particles enter cells upon contact is quite rapid, with reports of this occurring within in 1.2 seconds, followed by detection of viral DNA in the nucleus within 15 minutes *in vitro* [34]. Nevertheless, the speed with which vector dispersion occurs *in vivo* will depend on the route of administration, and thus may need to be considered [35].

*In vivo* analysis of AAV6.2FF transduction efficiency in the lungs of mice following intranasal administration revealed AAV6.2FF formulated with 0.001% PF-68 transduced 70.75% of lung epithelial cells, defined as CD45^-^ EpCAM^+^ GFP^+^, compared to 51.87% cells in mice administered AAV6.2FF formulated in PBS alone. Therefore, despite the observed decrease in infectivity in AAV6.2FF formulated with PF-68 after incubation at 37℃ for four or more hours, this did not translate to reduced efficacy *in vivo*, likely because the vector was only exposed to 37℃ for only a short period of time before it entered a lung epithelial cell. One reason why AAV6.2FF performed better *in vivo* when formulated with PF-68 could be because PF-68 is thought to prevent protein aggregation or precipitation upon shear stress or when exposed to air-water interface [18, 36, 37], which for AAV has been reported to alter biodistribution or immunogenicity of the vector [38], and surface adsorption during vector administration [18, 19], which may have resulted in less vector loss during the administration procedure.

At 55℃, vector formulations with or without PF-68 showed a similar decrease in infectivity after 30 minutes. Analysis of average luciferase expression over time revealed that infectivity was slightly more reduced in PF-68 containing vector; however, particle integrity data suggested the opposite. In any case, there were no statistically significant differences between the two buffer formulations at 55℃. One possible explanation for this discrepancy could be that at higher temperatures, PF-68 might have led to increased particle aggregation and surface adsorption due to changes in protein folding, or the effectiveness of this formulation drops at higher temperatures [39].

The starting titers were the same for AAV samples buffered in PBS or PBS supplemented with 0.001% PF-68 as they were derived from the same vector preparation. While there was no significant difference in infectivity of AAV samples buffered in PBS or PBS-PF-68 and stored at -80℃ (Fig. 2), there were significant differences when analyzed for particle integrity by qPCR (Fig. 3). This discrepancy may be due to the higher sensitivity of the qPCR assay.

AAV is typically stored and shipped at ultralow temperatures less than -60℃ as this has been shown to minimize chemical and physical degradation of AAV [15]. However, during manufacturing and clinical use, AAV vectors are exposed to freeze-thaw cycles as well as short-term exposures to room temperature (21℃) and refrigerated (4℃) conditions [18]. Freeze-thaws may cause several issues that can lead to decreased potency including, formation of crystalline excipients or air bubbles, cryo-concentration of excipients, exposure of vector to surfaces of ice, shifts in pH during freezing, or denaturation [20]. Moreover, many of these factors can be stochastic or variable in nature, which may have contributed to some of the variability in infectivity we observed between the different freeze-thaw cycles. A consequence of these events that occur during freeze-thaw is the release of small amounts of DNA from disrupted AAV capsids [40]. Indeed, we observed an increase in non-encapsidated or free viral DNA released from AAV6.2FF vectors buffered in PBS starting after as little as one freeze-thaw cycle; however, this was not observed when AAV6.2FF was formulated in PBS plus 0.001% PF-68. In fact, AAV6.2FF was relatively resistant to the effects of multiple freeze-thaws (up to 10 freeze-thaw cycles) when PF-68 was present. These results underpin the observations of other labs [16, 18, 20] and reinforce the importance of including a non-ionic surfactant such as PF-68 in AAV buffer formulations.

Some limitations of this study include the potential for transgene specific impacts on vector stability. It has previously been demonstrated that greater thermal instability is observed with shorter and oversized AAV genomes as heating can lead to structural changes impacting genome release [41]. Also, we did not evaluate the impact of pH changes or inclusion of a cryoprotectant (e.g., sucrose, sorbitol, or mannitol) on AAV6.2FF stability or infectivity, but this is something we will investigate in the future. Interestingly, a study by Barnes *et al.* concluded that the AAV6 serotype might be prone to disassembly, aggregation, and sensitive to freeze-thaws [41]. AAV6.2FF is derived from AAV6; however, the impacts of temperature and freeze-thaw on particle stability were largely mitigated by the inclusion of PF-68. Finally, it is unclear whether the results of this study can be extrapolated to other AAV serotypes; however, the fact that PF-68 is an increasingly common component in AAV buffer formulations suggests that the beneficial properties of PF-68 will extend to other AAV serotypes.

## Conclusions

We have shown that inclusion of PF-68 can prevent the degradation of AAV6.2FF vectors and preserve infectivity at a range of temperatures and freeze-thaw cycles, which could lead to improved shelf life and cold-chain requirements for shipping and storing AAV6.2FF vectors.

### Supplementary Information

**Supplementary Material 1.**

## Data Availability

No datasets were generated or analysed during the current study.

## Abbreviations

AAV: Adeno-associated virus
vg: vector genomes
ffLuc: firefly luciferase
eGFP: enhanced green fluorescent protein
PBS: phosphate buffered saline
PF-68: Pluronic F-68
IND: Investigational New Drug Application
FDA: Food and Drug Administration
EMA: European Medicines Agency
qPCR: quantitative PCR
F/T: freeze-thaw
