# Supplementary material for: Analysis of the impact of pluronic acid on the thermal stability and infectivity of AAV6.2FF
Source: BMC Biotechnol. 2024 Apr 25;24:22. doi: 10.1186/s12896-024-00853-6 (PMC11045451; doi:10.1186/s12896-024-00853-6)
Supplement: Supplementary file 1 — Supplementary Material 1. [file 12896_2024_853_MOESM1_ESM.pdf]

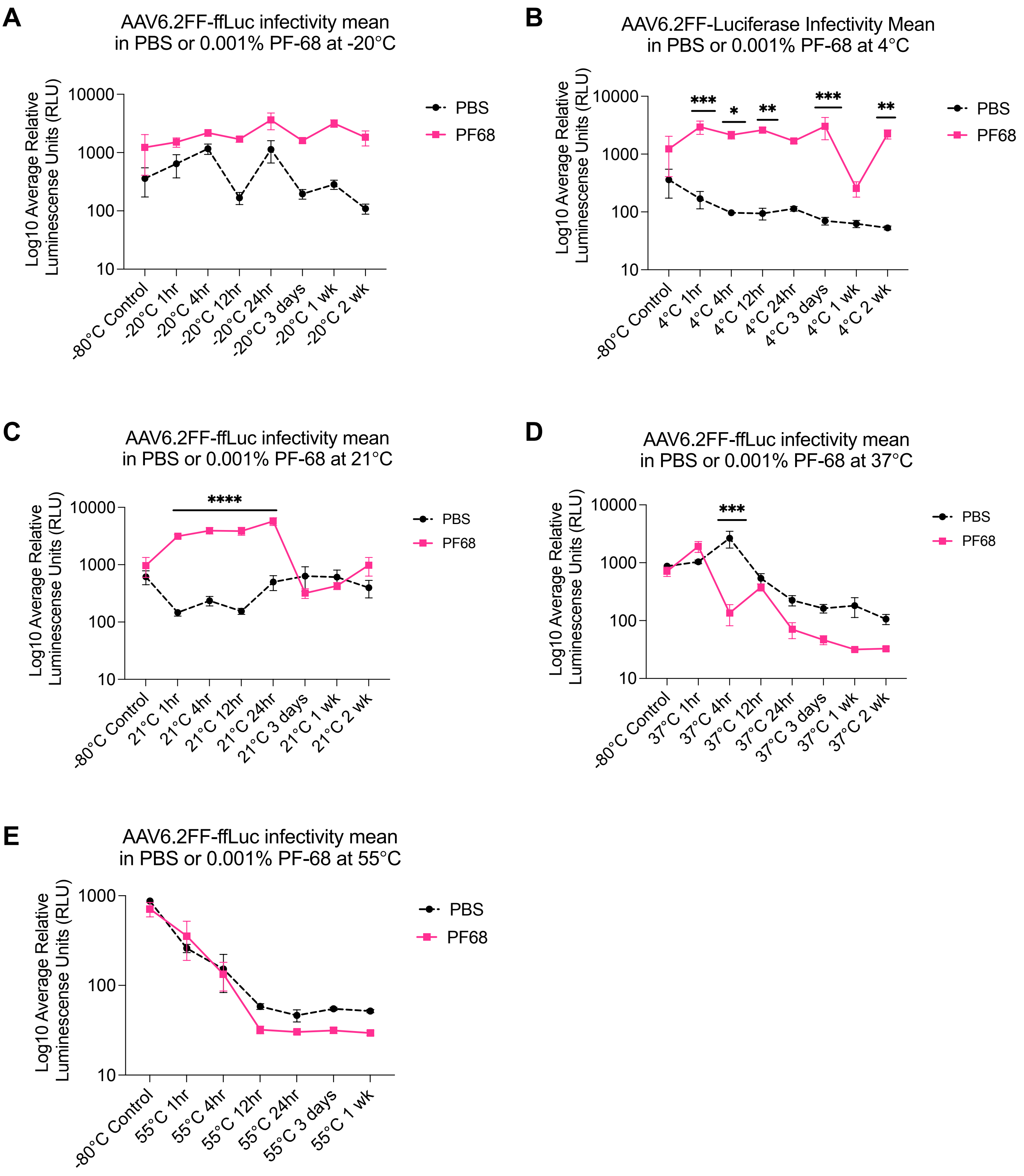


**Figure S1. Average AAV6.2FF-ffLuc infectivity with or without 0.001% PF-68 in different exposure conditions**. Aliquots of $1x10^9$ vg of AAV6.2FF-ffLuc formulated PBS or PBS supplemented with 0.001% PF-68 were exposed to a range of temperatures and durations. Following treatment, vector aliquots were applied to HEK293 cells and luciferase expression measured in relative luminescent units (RLU) 72 hours later. The conditions tested were as follows: (A) -20ºC, (B) 4ºC, (C) 21ºC, (D) 37ºC, or (E) 55ºC. Average RLU for each time point and buffer formulation are graphed.

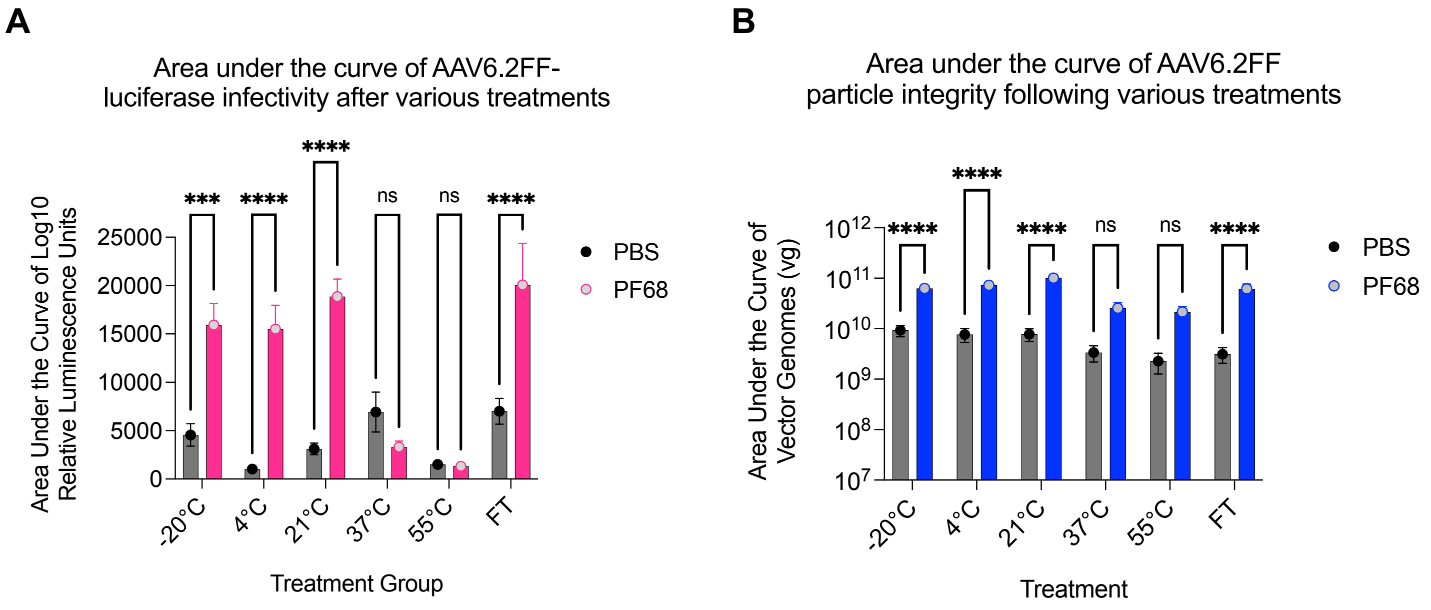


**Figure S2. Area under the curve of the luciferase infectivity and vector integrity measurements for each experimental treatment.**

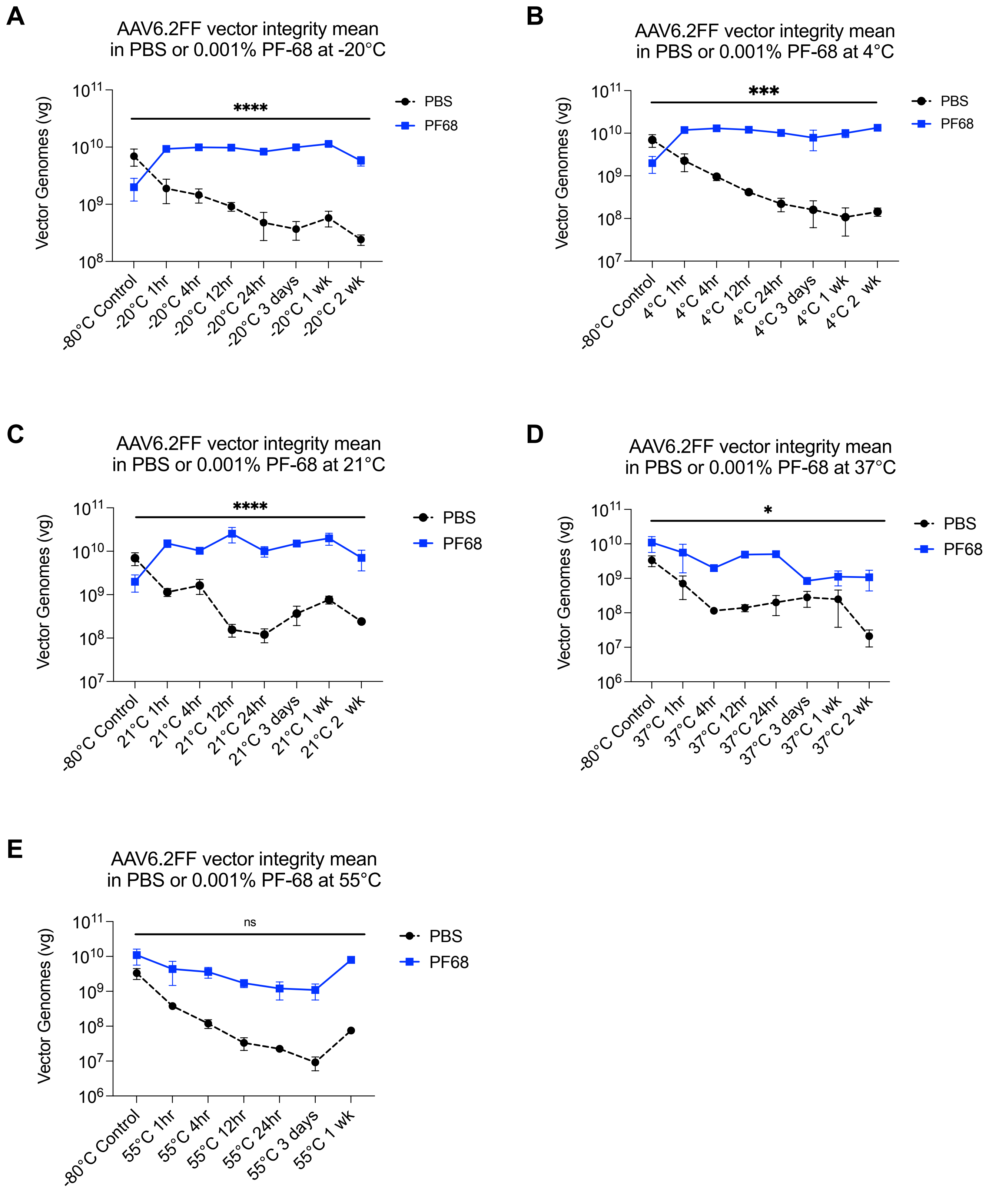


**Figure S3. Average vector genome copies of AAV6.2FF-ffLuc formulated in PBS or 0.001% PF-68 after exposure to different temperatures**. Aliquots of 1x10⁹ vg of AAV6.2FF-ffLuc formulated PBS or PBS supplemented with 0.001% PF-68 were exposed to a range of temperatures and durations. Following treatment, viral DNA was extracted and quantified by qPCR. The conditions tested were as follows: (A) -20ºC, (B) 4ºC, (C) 21ºC, (D) 37ºC, or (E) 55ºC. Average vector genome copies for each time point and buffer formulation are graphed.

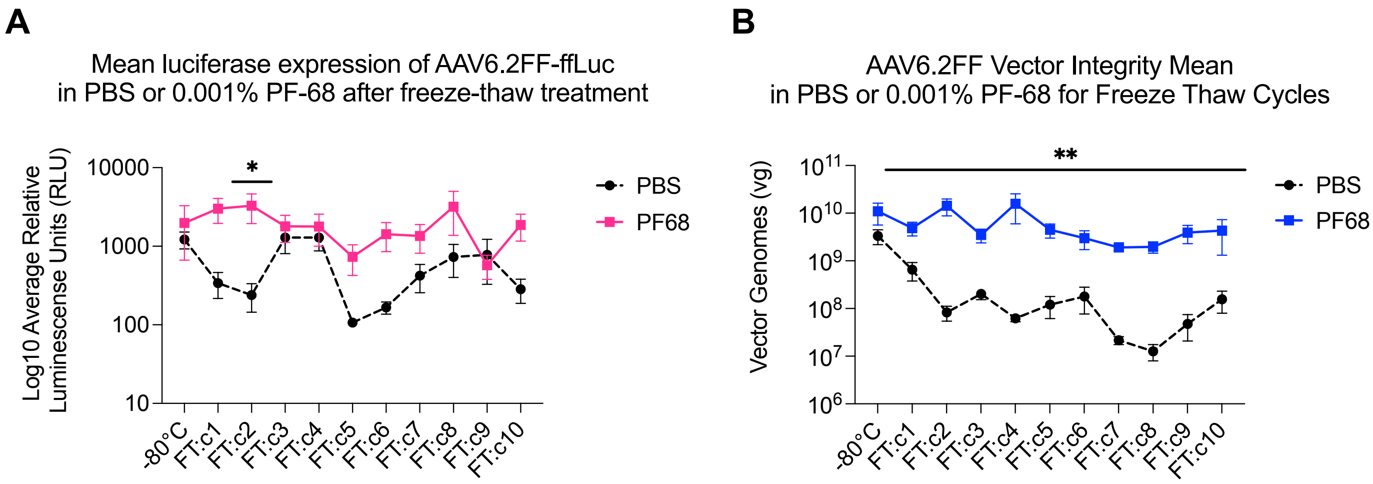


**Figure S4. Average infectivity and number of vector genomes of AAV6.2FF-ffLuc in different buffer formulations following repeated freeze-thaw.** Aliquots of $1x10^9$ vg of AAV6.2FF-ffLuc formulated PBS or PBS supplemented with 0.001% PF-68 were exposed to 1 to 10 cycles of freeze thaw after which the samples were either applied to HEK293 cells and luciferase reporter gene expression quantified 72 hours later (A), or viral DNA was extracted and quantified by qPCR (B). Average RLU (A) or vector genome copies (B) for each time point and buffer formulation are graphed.
